# Contentment Duration Mediates the Associations between Anxious Attachment Style and Psychological Distress

**DOI:** 10.3389/fpsyg.2017.00258

**Published:** 2017-02-22

**Authors:** Sin Man Ng, Wai Kai Hou

**Affiliations:** ^1^Department of Psychology, The Education University of Hong KongHong Kong, Hong Kong; ^2^Laboratory of Psychobiology of Emotion and Stress, The Education University of Hong KongHong Kong, Hong Kong; ^3^Centre for Psychosocial Health, The Education University of Hong KongHong Kong, Hong Kong

**Keywords:** adult attachment styles, anxiety, depression, intensity and duration of contentment, anxious and avoidant attachment

## Abstract

Relatively little is known about the emotional processes underlying the association between adult attachment styles and psychological distress. This study aims to examine the role of contentment in terms of intensity and duration in the positive associations between anxious and avoidant attachment styles and psychological distress. A sample of 284 Chinese university students completed a self-reported questionnaire on attachment styles, intensity and duration of contentment, and anxiety and depressive symptoms. Structural equation modeling revealed that duration of contentment mediated the positive associations of anxious attachment style with anxiety symptoms [β = 0.05, *p* = 0.004; BC 95% CI (0.02,0.11)] and depressive symptoms [β = 0.04, *p* = 0.03; BC 95% CI (0.003,0.09)], model fit: χ^2^(259) = 455.06, *p* < 0.001, CFI = 0.95, TLI = 0.94, RMSEA = 0.05, SRMR = 0.07. Participants with higher anxious attachment style were more likely to report shorter duration of contentment, which was, in turn, associated with higher anxiety and depressive symptoms. The results suggest a positive emotional pathway underlying the association between anxious attachment style and psychological distress. Implications based on the findings are discussed.

## Introduction

Adult attachment styles, namely anxious and avoidant attachment styles, have been found to impair development of emotional bonding in intimate relationships (Hazan and Shaver, 1987) and psychological functioning (Dozier et al., 2008). The positive attachment-distress link was reduced by trait mechanisms including dispositional hope (Shorey et al., 2003), dispositional empathy and forgiveness (Burnette et al., 2009), and self-esteem (Roberts et al., 1996). The link was enhanced by interpersonal mechanisms of lower perceived spousal support (Simpson et al., 2003) and lower perceived social rank (Irons and Gilbert, 2005).

Other possible mechanisms underlying the attachment-distress link await further investigation. Adult attachment theory suggests that attachment strategies could meet attachment needs through affective mechanisms (Mikulincer and Shaver, 2003, 2007). Positive emotions contribute to higher fulfillment of life (Fredrickson and Losada, 2005) and lower psychological distress (Davidson et al., 2010). Among discrete positive emotional states, contentment is a consistent component of happiness across cultures (Lu, 2001; Veenhoven, 2009). Contentment-associated reflective and broadening thoughts are suggested to contribute to higher psychological well-being (Izard, 2007, 2009, 2011). This study aims to investigate whether and how contentment as an affective mechanism modulates the positive associations between adult attachment styles and psychological distress.

### Attachment-Distress Link

The attachment theory (Bowlby, 1979) proposes that our relationship with caregiver(s) early in life forms working models of attachment. The working models shape representations of self and others that influence how we build up interpersonal relationships throughout the lifespan (Berlin et al., 2008). The working models could be revealed in self-reported anxious and avoidant attachment styles (Brennan et al., 1998; Fraley and Waller, 1998). Higher anxious attachment style is associated with the tendency of fearing others’ rejection and abandonment whereas higher avoidant attachment style is associated with the tendency of fearing intimacy and dependence on others (Hazan and Shaver, 1987; Brennan et al., 1998). Secure attachment styles, indicated by lower anxious and avoidant attachment styles, confers higher social competence and capacity for secure, supportive, and intimate relationships (Hazan and Shaver, 1987; Brennan et al., 1998). Attachment styles have been found to predict psychological distress among both psychiatric and non-clinical populations (Mickelson et al., 1997; Lopez and Brennan, 2000; Eng et al., 2001; Hankin et al., 2005; Mikulincer and Shaver, 2007; Dozier et al., 2008). Both anxious and avoidant attachment styles were positively associated with anxiety and depressive symptoms (Mikulincer and Orbach, 1995; Roberts et al., 1996; Mickelson et al., 1997; Zhang and Labouvie-Vief, 2004; Colonnesi et al., 2011).

### Affective Mechanism

The adult attachment theory suggests that affective regulatory strategies would be developed under the condition that significant others are unavailable or unresponsive when individuals are in need (Mikulincer et al., 2003). Anxious and avoidant attachment styles regulate hyperactivating and deactivating attachment strategies, respectively. To minimize distance from others, individuals with higher anxious attachment style tend to seek support and closeness intensively and be hypervigilant to potential social rejection threats, resulting in amplification of negative emotional responses and minimization of positive ones (i.e., hyperactivating strategies; Mikulincer et al., 2003). On the other hand, to minimize attachment need, individuals with higher avoidant attachment style tend to decline support and intimacy from others and rely on themselves, resulting in habitual suppression of emotions (i.e., deactivating strategies; Mikulincer et al., 2003). Therefore, individuals with different attachment styles might experience different emotional experiences due to their corresponding attachment strategies. Affective mechanisms could modulate the association between attachment styles and psychological distress.

Studies of adult attachment focus primarily on negative emotions (Gentzler and Kerns, 2006) notwithstanding the large body of literature on the links of positive emotions with lower psychological distress and higher psychological well-being (Fredrickson, 2013). According to the broaden-and-build theory (Fredrickson, 1998), positive emotional states such as joy, happiness, interest, contentment, and love broaden thought-action repertoires and enhance psychosocial resources for coping. Cross-sectional, longitudinal, and experience sampling evidence is available to show that anxious and avoidant attachment styles predict lower levels of positive emotions (Simpson, 1990; Magai et al., 1995; Torquati and Raffaelli, 2004). University students with higher self-reported anxious and avoidant attachment styles demonstrated more underestimation and minimization of positive emotional experiences relative to those with lower anxious and avoidant attachment styles (Gentzler and Kerns, 2006; Gentzler et al., 2010). It is also well established that positive emotions are inversely associated with anxiety and depressive symptoms (Headey et al., 1993; Watson et al., 1988; Davidson et al., 2010).

### Contentment

Among discrete positive emotional states, contentment has been suggested to be a culture-free and important component of happiness despite cross-cultural differences in positive emotional experiences (Lu, 2001; Lu and Gilmour, 2004; Miyamoto and Ma, 2011). Contentment is considered a frequently occurring positive emotional state in daily life (Fredrickson, 1998, 2013; Carstensen et al., 2000; Izard, 2011; Levenson, 2011). Contentment emerges if individuals appraise circumstances as cherished or satisfying (Fredrickson, 2013) and if personal and social resources match or exceed circumstantial demands (Shiota et al., 2006; Shiota and Kalat, 2012).

Contentment is closely associated with but distinctly different from joy and happiness. Joy, sometimes experienced spontaneously, results in higher behavioral activation, whereas contentment results in moderate behavioral activation through interactions with higher-ordered cognitions (Frijda, 1986; Izard, 2007, 2009, 2011). For instance, contented individuals demonstrated lower amplitude of body movement in walking compared with joyful individuals (Gross et al., 2012). Also, unlike joy, contentment does not emerge from circumstances that are appraised as unexpectedly good (Fredrickson, 2013). Contentment is also different from happiness although both states generate moderate behavioral activation (Lazarus, 1991). Happiness could indicate overall psychological well-being (Veenhoven, 2009; Fredrickson, 2013) and positive appraisals of life and affective experiences (Veenhoven, 2009), whereas contentment could contribute to well-being through building more refined and complex sense of self/priorities and broadened views of the self/world (Fredrickson, 1998, 2013).

The broaden-and-build theory further suggests that contentment creates an urge to savor and integrate present circumstances into new priorities and values (Fredrickson and Levenson, 1998; Fredrickson, 2013). Through these processes, contentment brings to individuals reflective and broadening thoughts that benefit adaptive behaviors (Fredrickson, 2000; Fredrickson and Branigan, 2005; Gross et al., 2012). Contentment can be measured both as a trait and as a state (Shiota et al., 2006). Dispositional contentment was inversely associated with anxious attachment style among university students (*N* = 108; Shiota et al., 2006). State contentment has also been associated with lower levels of anxiety and depressive symptoms (*N* = 203; Gilbert et al., 2008) and faster recovery from cardiovascular reactions to negative emotions (Fredrickson and Levenson, 1998; Fredrickson et al., 2000). Previous evidence suggests that the higher the levels of anxious and avoidant attachment styles, the lower contentment and the higher psychological distress one might experience. However, no studies to date have investigated the nature of associations among adult attachment styles, contentment, and psychological distress.

### The Present Study

The aim of this study is to examine the role of contentment in the associations between adult attachment styles and psychological distress. Two issues were taken into account for assessing contentment. First, intensity and duration are the two core features of most if not all emotional experiences (Sonnemans and Frijda, 1994; Frijda, 2007; Verduyn et al., 2011; Brans and Verduyn, 2014). Therefore, this study assessed both features in order to obtain a comprehensive understanding of contentment. Second, report of well-being in specific circumstances is less prone to recall biases compared with report of global well-being (Reis and Gable, 2000). We thus assessed intensity and duration of contentment with reference to a recent and significant experience of the participants.

Based on the adult attachment theory and the broaden-and-build theory, we predicted that anxious and avoidant attachment orientations would be associated with lower intensity and shorter duration of contentment and higher anxiety and depressive symptoms. We also expected that both intensity and duration of contentment would concurrently mediate the associations between anxious and avoidant attachment styles and anxiety and depressive symptoms. The two attachment styles would be associated with lower intensity and shorter duration of contentment and, in turn, higher anxiety and depressive symptoms. Giving the cross-sectional nature of this study, an alternative model with the mediating effects of the two attachment styles in the associations between contentment intensity and duration and psychological distress was tested.

## Materials and Methods

### Participants and Procedure

Upon obtaining Ethics Committee’s approval from the university, a recruitment notice was posted on the university’s intranet that is accessible to all students. Inclusion criteria were 18 years of age or older, Chinese ethnicity, and Cantonese fluency. Exclusion criterion was history of psychiatric or neurological disorder(s). Research staff checked whether the participants’ self-reported demographic information conformed with the inclusion and exclusion criteria. The data was collected between December 2012 and February 2013. A total of 284 students were fully apprised of the study by research staff, gave written informed consent, and self-administered a questionnaire set in an office room in the university. All participants received a HK$50 supermarket coupon (US$1 ≈ HK$7.80) upon completion of the questionnaire. The 284 participants ranged in age between 18 and 37 years (*M* = 21.75, *SD* = 2.43, median = 21.76); 234 (82.4%) were female and three (1.1%) were married. Sixty-two participants (21.8%) reported a part-time employment.

### Measures

#### Adult Attachment Styles

The Chinese version of the 9-item Experiences in Close Relationships – Relationship Structures questionnaire (ECRRS; Mallinckrodt and Wang, 2004; Fraley et al., 2011a) assessed anxious and avoidant attachment styles. Three items measured anxious attachment style while the remaining six items measured avoidant attachment style. Participants were asked to specify one close social partner and responded to the items on a 7-point scale (1 = *strongly disagree*, 7 = *strongly agree*). Higher scores indicated more anxious/avoidant attachment. Sample items are “*I’m afraid that this person may abandon me*” (anxious attachment orientation) and “*I talk things over with this person*” (avoidant attachment orientation). The anxious and avoidant attachment subscales have been found to be reliable (Cronbach’s α = 0.80–0.88) and validly associated with personalities and psychological outcome variables among Chinese (Mallinckrodt and Wang, 2004). Cronbach’s alphas for the anxious and avoidant attachment styles in the current administration were both 0.82 in the current administration.

#### Intensity and Duration of Contentment

No validated English and Chinese measures were available for assessing perceived intensity and duration of contentment at the time of study. The research team drafted 10 Chinese items to assess perceived intensity and duration of contentment (five items each) in a recent experience. The items were translated into English by a trained bilingual translator and then back-translated by a naïve second translator. The research team adopted a combined etic-emic approach in order to achieve semantic and conceptual equivalence of the original and the back-translated versions. Discrepancies in the two versions were resolved by joint meetings between the translators and the research team and when necessary by reiteration of the translation process. The instruction read “*Please recall a recent situation that made you feel contented, and indicate the extent to which each statement describes the experience.*” Participants rated each item on a 5-point scale (0 = *does not describe me at all*, 4 = *describe me very well*) (**Table 1**). Exploratory factor analysis (EFA) with promax rotation was conducted on the items. The latent root criterion and the scree plot suggested a two-factor model (61.86% of the total observed variance) of intensity (five items) and duration (five items). The factor loadings of the items were 0.45–0.77 (intensity) and 0.52–0.94 (duration) respectively. Cronbach’s alphas in the current administration were 0.77 for intensity and 0.87 for duration.

**Table 1:** Scale of intensity and duration of contentment.

Item	
I1	The contented feelings got out of control.
I2	I felt contented, finding it hard to feel about much else.
I3	The contentment was very overpowering, such that I become quite spontaneous.
I4	The contentment was so profound; it was difficult to think about anything else.
I5	I have strong feelings of contentment.
D1	The feelings of contentment were short-lived. ^a^
D2	I could quickly erase the feelings of contentment. ^a^
D3	I was quickly calm after being contented, easily regaining my composure. ^a^
D4	The feelings of contentment stayed with me for prolonged time.
D5	The contentment only lasted a few moments. ^a^

^a^*Reverse scored; I, intensity of contentment; D, duration of contentment*.

#### State-Trait Anxiety Inventory – State Version

The Chinese version of the 20-item state version of State-Trait Anxiety Inventory (STAI; Shek, 1988; Spielberger, 1983) assessed anxiety symptoms in the past 2 weeks on a 4-point scale (1 = *almost never*, 4 = *almost always*). A summed score was used (range = 20–80). The Chinese version has been demonstrated to be reliable in the validation study (Cronbach’s α = 0.90; Shek, 1988) and in the current administration (Cronbach’s α = 0.93).

#### Beck Depression Inventory-II

The Chinese version of the 21-item Beck Depression Inventory-II (BDI-II; Beck et al., 1996; Byrne et al., 2004) was used to assess depressive symptoms in the past 2 weeks on a 4-point scale (e.g., 0 = *I do not feel sad*, 1 = *I feel sad*, 2 = *I am sad all the time*, 3 = *I am so sad or unhappy that I can’t stand it*) (range = 0–63). The Chinese version has demonstrated high internal consistency among Chinese people (Cronbach’s α > 0.91; Byrne et al., 2004). Cronbach’s alpha was 0.88 in the current administration.

### Analytic Plan

Multiple imputation was used to replace the missing data (0.4%) using SPSS (Version 21; SPSS Inc., Chicago, IL, USA). A set of plausible values that represented the uncertainty of the missing data was imputed (Rubin, 2004). Correlation analysis and Mann–Whitney *U* tests were used to identify demographic characteristics that were correlated with the study variables. Structural equation modeling (SEM) with maximum likelihood was conducted to test the mediation models (AMOS version 17; Arbuckle, 2008). Parcels of measured variables were used to indicate latent variables of avoidant attachment style, anxiety symptoms, and depressive symptoms. To perform the item parceling, EFA with oblique rotation were conducted on the items of each of the above variables. Items were assigned to their corresponding parcels in descending order based on factor loadings (Coffman and MacCallum, 2005).

A structural equation model was constructed to examine the nature of associations of anxious and avoidant attachment styles with intensity and shorter duration of contentment and anxiety and depressive symptoms. Anxiety and depressive symptoms were dependent variables, anxious and avoidant attachment styles were independent variables whereas intensity and duration of contentment were mediating variables in the hypothesized conceptual model. All study variables were latent variables. Error variances of the two dependent variables were correlated to account for unmeasured shared sources of variances (Kline, 2011). To construct a parsimonious model with better model fit, insignificant paths were constrained to 0. The parsimonious model was reported if (1) chi-square different tests did not reveal significant difference of it with the original one and (2) the model fit of it was better than that of the original one. Bootstrapping techniques were used to test the direct and indirect effects in the mediation models (Shrout and Bolger, 2002). A total of 5000 bootstrap samples were derived from the original data (*N* = 284) in order to generate bias-corrected 95% confidence intervals (BC 95% CI) as accurate tests of the mediating effects in the current modest sample size (MacKinnon et al., 2002). The mediating effects were supported if the indirect effects were significant at *p* < 0.05 and the BC 95% CI did not include 0 (Shrout and Bolger, 2002). The chi-square statistics, Comparative Fit Index (CFI), Tucker-Lewis Index (TLI), root mean square error of approximation (RMSEA), and standardized root mean square residual (SRMR) were used to evaluate data-model fit (Hu and Bentler, 1999). The model was accepted if CFI and TLI > 0.90, SRMR and RMSEA < 0.08 (Browne and Cudeck, 1993; Hu and Bentler, 1999).

In the alternative model, the independent variables (anxious and avoidant attachment styles) and mediating variables (intensity and duration of contentment) were swapped. The Akaike Information Criterion (AIC) and Bayes Information Criterion (BIC) were used to compare the hypothesized model with the alternative model (Kline, 2011). The model with lower AIC and BIC provided a better fit to the data.

## Results

### Descriptive Statistics

Means, standard deviations, and 0-order correlations of the study variables are summarized in **Table 2**. Gender (0 = females, 1 = males) was correlated with the study variables (*r* = 0.13–0.28, *p* < 0.001–0.07) and was thus included as an exogenous variable in all models. Age, education level, and family income (*r* = 0.06–0.92) were not significantly correlated with the study variables. The close social partners in ECRRS that the participants chose were parent (110; 38.7%), friend (73; 25.7%), partner (65; 22.9%), sibling (32; 11.3%), and grandparent or other family member (4; 1.4%).

**Table 2:** Descriptive statistics and 0-order correlations of the study variables.

	Mean	*SD*	Exact range	Possible range	1	2	3	4	5	6
(1) Anxious attachment style	9.38	4.90	3–21	3–21	-	0.02	0.03	-0.19^∗∗^	0.23^∗∗∗^	0.27^∗∗∗^
(2) Avoidant attachment style	17.76	6.48	6–39	6–42		-	-0.04	-0.002	0.13^∗^	0.08
(3) Contentment – Intensity	7.20	3.47	0–18	0–20			-	0.36^∗∗∗^	-0.03	0.05
(4) Contentment – Duration	9.89	4.20	0–20	0–20				-	-0.19^∗∗^	-0.14^∗^
(5) Anxiety symptoms	44.36	10.71	22–79	20–80					-	0.56^∗∗∗^
(6) Depressive symptoms	12.34	8.42	0–50	0–63						-

*^∗^*p* < 0.05, ^∗∗^*p* < 0.01, ^∗∗∗^*p* < 0.001*.

### Model Specification

**FIGURE 1:**
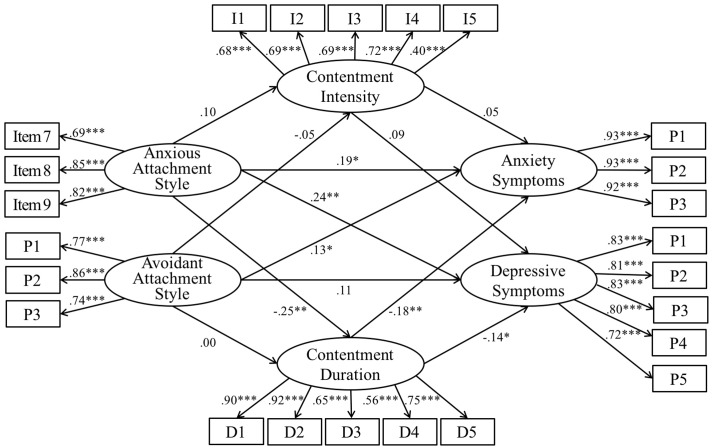
**Standardized estimates of the measurement model of the hypothesized model**. I = Intensity; D = Duration; P = Parcel. The items under each parcel, standardized regression coefficients of the error terms and covariate are not shown. ^∗^*p* < 0.05, ^∗∗^*p* < 0.01, ^∗∗∗^*p* < 0.001.

**FIGURE 2:**
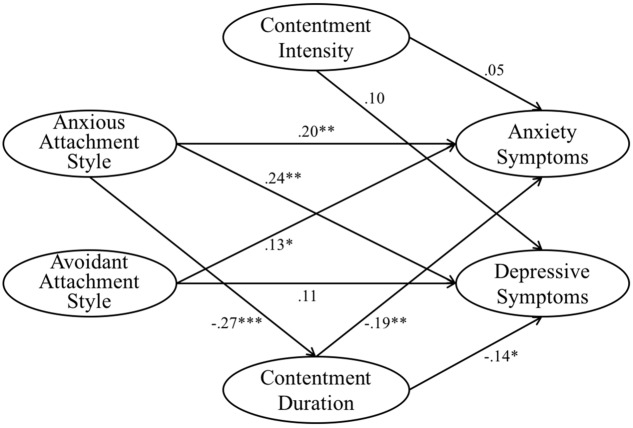
**Standardized estimates of the final mediation model of anxious and avoidant attchment styles, intensity and duration of contentment, and anxiety and depressive symptoms**. Standardized regression coefficients of the error terms, measured variables, and covariate are not shown. ^∗^*p* < 0.05, ^∗∗^*p* < 0.01, ^∗∗∗^*p* < 0.001.

The measurement model and the structural model were estimated sequentially (Anderson and Gerbing, 1988). Factor loadings of the measured variables on the latent variables were all significant (*p* < 0.001) (**Figure 1**). The model demonstrated good data-model fit: χ^2^(256) = 452.55, *p* < 0.001, CFI = 0.95, TLI = 0.94, RMSEA = 0.05, SRMR = 0.07. The paths from anxious attachment style to intensity of contentment (β = 0.10, *p* = 0.21) and from avoidant attachment style to intensity and duration of contentment (β = -0.05–0.00, *p* = 0.53–0.99) were not significant and thus constrained to 0. The model with constrained insignificant paths was not significantly different from the original one [Δχ^2^(3) = 2.51, *p* = 0.47] and demonstrated improved fit indices. The final model (**Figure 2**) demonstrated good data-model fit: χ^2^(259) = 455.06, *p* < 0.001, CFI = 0.95, TLI = 0.94, RMSEA = 0.05, SRMR = 0.07. The alternative model also demonstrated comparable data-model fit: χ^2^(256) = 452.67, *p* < 0.001, CFI = 0.95, TLI = 0.94, RMSEA = 0.05, SRMR = 0.07. However, the higher AIC and BIC of the alternative model (AIC = 590.67, BIC = 842.44) suggested that it provided a poorer fit to the data than the hypothesized model (AIC = 590.55, BIC = 842.33).

### Mediating Effects

In the hypothesized model, the total effects of anxious attachment style on anxiety symptoms (β = 0.25, *p* = 0.001) and depressive symptoms (β = 0.28, *p* < 0.001) were significant. Anxious attachment style was inversely associated with duration of contentment (β = -0.27, *p* < 0.001). Duration of contentment was inversely associated with anxiety symptoms (β = -0.19, *p* = 0.008) and depressive symptoms (β = -0.14, *p* = 0.05). Indirect effect of anxious attachment style on anxiety symptoms (β = 0.05, *p* = 0.004) and depressive symptoms (β = 0.04, *p* = 0.03) through duration of contentment were significant; the bootstrap BC 95% CI (0.003–0.02,0.09–0.11) did not include 0. Duration of contentment partially mediated the associations of anxious attachment style with anxiety and depressive symptoms.

## Discussion

The structural equation model showed that anxious attachment style was inversely associated with duration of contentment and positively associated with anxiety and depressive symptoms, consistent with previous evidence (e.g., Dozier et al., 2008). Contrary to our expectation, duration but not intensity of contentment was inversely associated with both anxiety and depressive symptoms. Also, only duration of contentment partially mediated the positive associations of anxious attachment style with anxiety and depressive symptoms (*Hypothesis 2*). The findings suggest that individuals with higher anxious attachment style are more likely to report shorter duration of contentment, which is, in turn, associated with higher anxiety and depressive symptoms. The alternative model with anxious and avoidant attachment styles as mediators between intensity and duration of contentment and anxiety and depressive symptoms demonstrated worse data-model fit. The result was consistent with the attachment theory (Bowlby, 1979), which suggests that attachment styles represent internal attachment model that was formed early in life. Attachment styles have been found to be relatively stable over time (Fraley, 2002; Fraley et al., 2011b). Therefore, conceptually attachment styles emerge prior to subjective experience of contentment than the other way round.

Discrete emotional states like contentment could show variable intensity and duration, which are interrelated but distinct dimensions of positive emotional states (Scherer and Wallbott, 1994; Sonnemans and Frijda, 1994; Verduyn et al., 2009a,b; Verduyn et al., 2012a). Although intensity and duration are important dimensions of positive emotional states, very few studies have examined subjective ratings of both dimensions and their associations with psychological functioning. In addition, most existing studies investigated intensity and/or duration of generic positive affect or discrete positive emotional states other than contentment (e.g., joy; Verduyn et al., 2011, 2012b). Contentment is suggested to have adaptive significance in its own right despite its overlaps with other positive emotional states (Frijda, 1986; Fredrickson, 1998, 2013; Gross et al., 2012).

Consistent with previous studies (Sonnemans and Frijda, 1994; Schimmack, 2003; Waugh et al., 2010), this study found that intensity and duration of contentment were positively associated with each other. Besides, the present findings demonstrated significant associations of duration of contentment, but not intensity of contentment, with anxiety and depressive symptoms. More intense positive emotions have been found to predict lower levels of anxiety symptoms among older adults (Cook et al., 2004). Also, intensity and duration of other positive emotional states (e.g., joy) have been found to jointly impact cognitive processes of emotions including self-distancing from emotional experiences (Verduyn et al., 2012b). However, in the present study, we assessed contentment instead of generic emotions. This might explain why the associations between intensity of contentment with anxiety and depressive symptoms were insignificant. The inverse associations between duration of contentment and anxiety and depressive symptoms could be due to longer time on building psychosocial resources through one’s broadened thought-action repertoires (Fredrickson, 1998, 2013). The resource building process could reduce the risk of developing psychological distress. In addition, it takes time for contentment-related savoring and integrating process to broaden thoughts and build up well-being (Fredrickson, 2013), so duration but not intensity of contentment impacts anxiety and depressive symptoms. The findings of the current study suggest that within contentment, duration could have higher adaptive utility relative to intensity.

This study also highlights the role of duration but not intensity of contentment in the associations between anxious attachment style and psychological distress. Individuals with higher anxious attachment style have been suggested to use hyperactivating strategies that exacerbate negative emotional states (Mikulincer et al., 2003; Mikulincer and Shaver, 2008). Although they could experience positive emotions in the initial stage of positive events, their higher anxious attachment style contributes to negative thoughts about the current and previous experiences, which in turn transforms the naturally occurring positive emotions into neutral or negative ones (Shaver and Mikulincer, 2008). Positive emotional episode might also be shorter due to emergence of thoughts that are related to negative emotions (Verduyn et al., 2011). This explained the inverse association between anxious attachment style and duration of contentment in this study. Anxious attachment style could reduce the possibility of forming savoring and integrating process through the contented emotional experience (Fredrickson, 2013). Longer duration of contentment could interact with the higher order cognition and form positive emotional schema (Izard, 2011) and counteract the habitual attachment-related hyperactivating strategy during positive experience. Therefore, duration of contentment mediated the link between anxious attachment style and anxiety and depressive symptoms in the present study. This study is one of the first to provide empirical evidence on buffering impact of duration of contentment in the positive association between anxious attachment style and psychological distress.

Avoidant attachment style was not significantly associated with intensity and duration of contentment and depressive symptoms. Individuals with higher avoidant attachment style tend to use deactivating strategies to block threat-related emotional experiences (Mikulincer et al., 2003). Higher deactivating strategies also contribute to dismissal of positive emotional experiences that promote interpersonal closeness and investment in interpersonal relationships (Cassidy, 1994; Mikulincer and Shaver, 2008). There is evidence showing that higher avoidant attachment style conferred lower levels of emotional experiences and higher tendency to suppress emotional expressions (Kerr et al., 2003). Moreover, inconsistent findings have been identified between avoidant attachment style and psychological distress (Mikulincer and Shaver, 2007). Because of the tendency to suppress emotional experiences among high-avoidant individuals, self-report measures might not adequately assess their depressive symptoms. Future studies could use indirect measures such as peer reports and physiological indices of emotions in order to replicate and extend the models in this study (Edelstein and Shaver, 2004).

### Limitations

Several limitations warranted cautions. First, this study was conducted in a convenient non-clinical sample of Chinese university students (Female participants: 82.4%). Gender differences might exist in emotional experiences (Nolen-Hoeksema, 2001; Fischer et al., 2004; Chentsova-Dutton and Tsai, 2007; Linley et al., 2015). For example, women were found to report higher frequency of joy while men reported higher frequency of pride (Brebner, 2003). Although gender was included as an exogenous variable to control for its effect, representativeness and generalizability of the present findings should be tested among people with different sociodemographic backgrounds. Second, cross-sectional design could have limited interpretation of the directions of associations among the study variables, although the alternative model that we tested was not founded upon conceptual knowledge (i.e., momentary contentment occurs prior to longstanding attachment styles) and demonstrated poorer data-model fit relative to our theory-driven hypothesized model. Third, the present analyses did not account for participants’ emotional states at the time of survey, which could inadvertently increase the chance of retrospective recall biases (Keuler and Safer, 1998). Fourth, reliability of the psychometric instrument of contentment should be confirmed in different populations, although the scales were found to be reliable and validly associated with other psychosocial variables in the present study.

## Conclusion

Notwithstanding these limitations, this study offers one of the first empirical evidence on the role of duration of contentment in the positive association between anxious attachment style and distress. Our findings also highlight the importance of up-regulating contentment in order to reduce the risk of poorer adjustment among adults. Higher positive emotions including contentment have been found to be increased by training on mindfulness (Jimenez et al., 2010), savoring strategies (Quoidbach et al., 2010), and awareness of positive emotional experiences (Fredrickson, 2000). Among these interventions, upregulation of contentment could be a core component for individuals with higher levels of anxious attachment style. They could be instructed to increase, intensify, and prolong contentment by thinking over the events triggered contentment or during experiencing those positive events (Hurley and Kwon, 2012). In addition, individuals could be instructed not to inhibit or reject thoughts, feelings, and sensations related to contentment before being encouraged to processes these components of contentment thoroughly (Hayes et al., 2006).

## Ethics Statement

The study was conducted upon obtaining approval from Human Research Ethics Committee, The Education University of Hong Kong (formerly The Hong Kong Institute of Education). After being fully apprised of the study, the participants gave written informed consent and self-administered the questionnaire.

## Author Contributions

SN and WH contributed to data collection and analysis, formulation of ideas, and write-up of the manuscript.

## Conflict of Interest Statement

The authors declare that the research was conducted in the absence of any commercial or financial relationships that could be construed as a potential conflict of interest. The reviewer VC and handling Editor declared their shared affiliation, and the handling Editor states that the process nevertheless met the standards of a fair and objective review.
